#  Water-filtered infrared-A (wIRA) overcomes swallowing disorders and hypersalivation – a case report

**DOI:** 10.3205/000252

**Published:** 2017-08-03

**Authors:** Gerd Hoffmann

**Affiliations:** 1 Institute of Sports Sciences, Johann Wolfgang Goethe University, Frankfurt/Main, Germany

**Keywords:** water-filtered infrared-A, wIRA, thermal effects, non-thermal effects, reduction of hypersalivation, reduction of mucus, reduction of hypersecretion, tissue temperature, tissue perfusion, tissue oxygen partial pressure, regeneration, quality of life, otorhinolaryngology, ENT medicine, oncology, physical medicine

## Abstract

**Case description:** A patient with a Barrett oesophageal carcinoma and a resection of the oesophagus with gastric pull-up developed swallowing disorders 6 years and 2 months after the operation. Within 1 year and 7 months two recurrences of the tumor at the anastomosis were found and treated with combined chemoradiotherapy or chemotherapy respectively. 7 years and 9 months after the operation local tumor masses and destruction were present with no ability to orally drink or eat (full feeding by jejunal PEG tube): quality of life was poor, as saliva and mucus were very viscous (pulling filaments) and could not be swallowed and had to be spat out throughout the day and night resulting in short periods of sleep (awaking from the necessity to spit out). In total the situation was interpreted more as a problem related to a feeling of choking (with food or fluid) in the sense of a functional dysphagia rather than as a swallowing disorder from a structural stenosis.

At that time acetylcysteine (2 times 200 mg per day, given via the PEG tube) and irradiation with water-filtered infrared-A (wIRA), a special form of heat radiation, of the ventral part of the neck and the thorax were added to the therapy. Within 1 day with acetylcysteine saliva and mucus became less viscous. Within 2 days with wIRA (one day with 4 to 5 hours with irradiation with wIRA at home) salivation decreased markedly and quality of life clearly improved: For the first time the patient slept without interruption and without the need for sleep-inducing medication. After 5 days with wIRA the patient could eat his first soft dumpling although drinking of fluids was still not possible. After 2½ weeks with wIRA the patient could eat his first minced schnitzel (escalope).

Following the commencement of wIRA (with typically approximately 90–150 minutes irradiation with wIRA per day) the patient had 8 months with good quality of life with only small amounts of liquid saliva and mucus and without the necessity to spit out. During this period the patient was able to sleep during the night.

**Discussion:** The main physiological effects of water-filtered infrared-A (wIRA) are: wIRA increases tissue temperature, tissue oxygen partial pressure and tissue perfusion markedly.

The five main clinical effects of wIRA are: wIRA decreases pain, inflammation and exudation/hypersecretion, and promotes infection defense and regeneration, all in a cross-indication manner. Therefore there is a wide range of indications for wIRA.

The effects of wIRA are based on both its thermal effects (relying on transfer of heat energy) and thermic effects (temperature-dependent effects, occurring together with temperature changes) as well as on non-thermal and temperature-independent effects like direct effects on cells, cell structures or cell substances.

**Conclusion:** Besides in a variety of other indications for wIRA, in cases of swallowing disorders (functional dysphagia) and hypersalivation or hypersecretion of mucus the use of wIRA should be considered as part of the treatment regime for improving a patient’s quality of life.

## Zusammenfassung

**Fallbeschreibung:** Ein Patient mit einem Barett-Ösophagus-Karzinom und Resektion des Ösophagus mit Magenhochzug-Operation bekam 6 Jahre und 2 Monate nach der Operation Schluckbeschwerden. Innerhalb von 1 Jahr und 7 Monaten wurden zwei Tumorrezidive an der Anastomose gefunden und mit kombinierter Radio-Chemo-Therapie bzw. Chemotherapie behandelt. 7 Jahre und 9 Monate nach der Operation bestanden lokale Tumormassen und Destruktion ohne Möglichkeit, etwas oral zu trinken oder zu essen (Ernährung vollständig über eine jejunale PEG-Sonde): die Lebensqualität war schlecht, da Speichel und Schleim sehr zäh (fädenziehend) waren und nicht geschluckt werden konnten, sondern Tag und Nacht ausgespuckt werden mussten, so dass nachts keine längere Zeitspanne geschlafen werden konnte (durch die Notwendigkeit auszuspucken jeweils wachwerdend). Insgesamt wurde die Situation mehr als eine funktionelle Schluckstörung/Dysphagie denn als ein strukturelles stenotisches Problem gewertet.

Zu diesem Zeitpunkt wurden Acetylcystein (zweimal täglich 200 mg über die PEG-Sonde) und Bestrahlung mit wassergefiltertem Infrarot A (wIRA), eine spezielle Form der Wärmestrahlung, der ventralen Seite des Halses und des Thorax zur Therapie dazugenommen. Innerhalb eines Tages mit Acetylcystein wurden Speichel und Schleim weniger zäh. Innerhalb von 2 Tagen mit wIRA (ein Tag mit 4–5 Stunden Bestrahlung mit wIRA zuhause) nahm die Speichelbildung deutlich ab und die Lebensqualität verbesserte sich ganz offensichtlich: zum ersten Mal schlief der Patient ohne Unterbrechung und ohne Schlafmittel. Nach 5 Tagen mit wIRA konnte der Patient seinen ersten weichen Kloß essen, Trinken von Flüssigkeiten war zu diesem Zeitpunkt noch nicht wieder möglich. Nach 2½ Wochen mit wIRA konnte der Patient sein erstes kleingeschnittenes Schnitzel essen.

Ab dem Beginn mit wIRA hatte der Patient 8 Monate mit guter Lebensqualität mit nur kleinen Mengen an flüssigem Speichel und Schleim und ohne Notwendigkeit auszuspucken (mit typischerweise ungefähr 90–150 Minuten Bestrahlung mit wIRA täglich). In dieser Zeitspanne konnte der Patient während der Nacht schlafen.

**Diskussion:** Die physiologischen Haupteffekte von wassergefiltertem Infrarot A (wIRA) sind: wIRA steigert die Gewebetemperatur, den Gewebe-Sauerstoffpartialdruck und die Gewebedurchblutung deutlich.

Die fünf klinischen Hauptwirkungen von wIRA sind: wIRA mindert Schmerzen, Entzündung und Exsudation/Hypersekretion, und fördert Infektionsabwehr und Regeneration, alles indikationsübergreifend. Entsprechend gibt es eine Vielzahl von klinischen Indikationen für wIRA.

Die Effekte von wIRA beruhen sowohl auf seinen thermischen (auf der Übertragung von Wärmeenergie basierenden) und temperaturabhängigen (mit Temperaturänderung auftretenden) als auch auf seinen nicht-thermischen und temperaturunabhängigen Effekten wie direkten Effekten auf Zellen, Zellstrukturen oder Zellsubstanzen.

**Schlussfolgerung: **Außer bei einer Vielzahl anderer Indikationen für wIRA sollte die Anwendung von wIRA bei Schluckstörungen (funktioneller Dysphagie, Verschlucken) und vermehrter Speichelbildung oder Hypersekretion von Schleim als Teil der Behandlung zur Verbesserung der Lebensqualität des Patienten erwogen werden.

## Case description

A 54-year-old male patient was diagnosed with Barrett oesophageal carcinoma (pT1 M0 N0 R0). In the same month the oesophagus was resected with gastric pull-up operation in a university clinic which kept medical attendance over the whole time.

5 years after the operation hoarseness appeared being interpreted as paralysis of the recurrent nerve, although this was surprisingly at all times not thought to be related to the basic disease by the university hospital. (From the author’s point of view the hoarseness can be interpreted as first sign of a recurrence of the tumor.)

6 years and 2 months after the operation swallowing disorders started to occur. 6 years and 4 months after the operation a recurrence of the tumor at the anastomosis was diagnosed and treated during the following 3 months with a combined chemoradiotherapy. 

7 years and 4 months after the operation a second local stenosing recurrence of the tumor at the anastomosis was diagnosed and subsequently treated for 4 months with chemotherapy. Repeated ballon dilatations of the oesophagus were carried out in order to treat pronounced swallowing disorders. 7 years and 7 months after the operation, an implanted oesophageal stent was not tolerated (local pain) and had to be removed within a week. A second attempt to position an oesophageal stent led to a respiratory arrest and a revival situation. Percutaneous endoscopic gastrostomy (PEG) was performed to position a jejunal PEG tube for feeding, bypassing the swallow disturbances. A diagnostic swallow of contrast material induced a choking response resulting in an atypical aspiration pneumonia. 

7 years and 9 months after the operation local tumor masses and destruction (with e.g. erosion of the seventh cervical vertebra body, already found earlier) and suspicion of lung metastases (later confirmed) were present with no ability to orally drink or eat (full feeding by jejunal PEG tube): quality of life was poor, as saliva and mucus were very viscous (pulling filaments) and could not be swallowed and had to be spat out throughout the day and night resulting in short periods of sleep (awaking from the necessity to spit out). In total (taking into account several aspects including clinical observation of swallowing acts and the increase of amount of saliva spat out within a short time span) the situation was interpreted by the author, in contrast to the university hospital, more as a problem related to a feeling of choking (with food or fluid) in the sense of a functional dysphagia rather than as a swallowing disorder from a structural stenosis.

**Figure 1:**
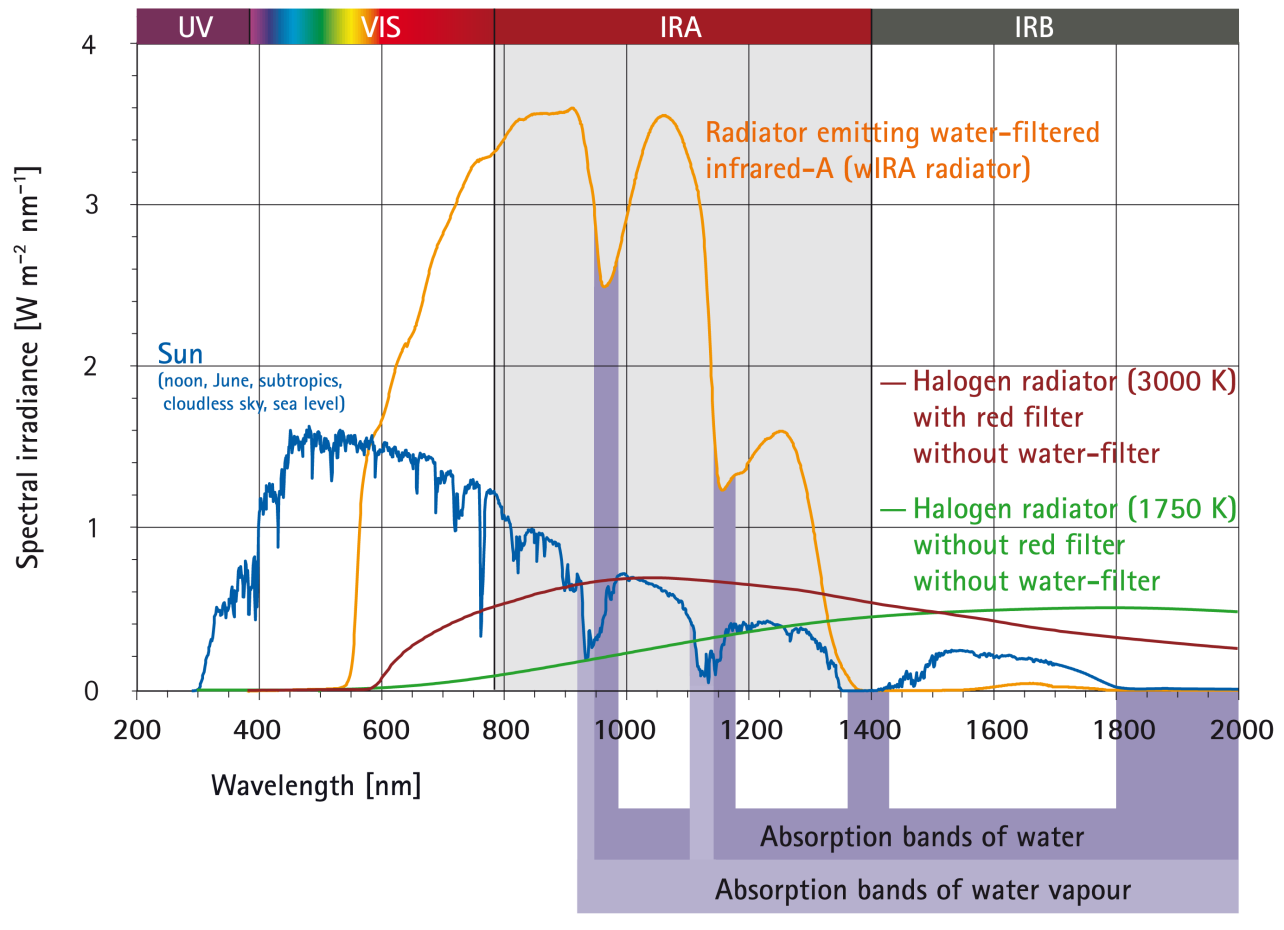
Comparison of the spectra of a radiator with water-filtered infrared-A (wIRA) and of the sun measured under a cloudless sky at noon in June at sea level in the subtropics and of two different halogen radiators without water-filter for therapeutic and wellness applications (with kind permission of Dr. Helmut Piazena, Charité Berlin; from [13]). The presented spectral irradiances of the wIRA radiator and of the two different halogen radiators cause the same skin surface temperature rise in humans (temperature-related equivalence of the irradiations). The presented solar irradiance is near the maximum possible value in the subtropics at noon in midsummer on the surface of the Earth at sea level with cloudless sky. The relations between the four presented spectra are therefore realistic. A typical wIRA radiator emits no ultraviolet radiation (UV) and almost no infrared-B and infrared-C radiation (less than 0.5% compared to 50–80% infrared-B and infrared-C in conventional infrared radiators without water-filter) (details in [13]).

**Figure 2:**
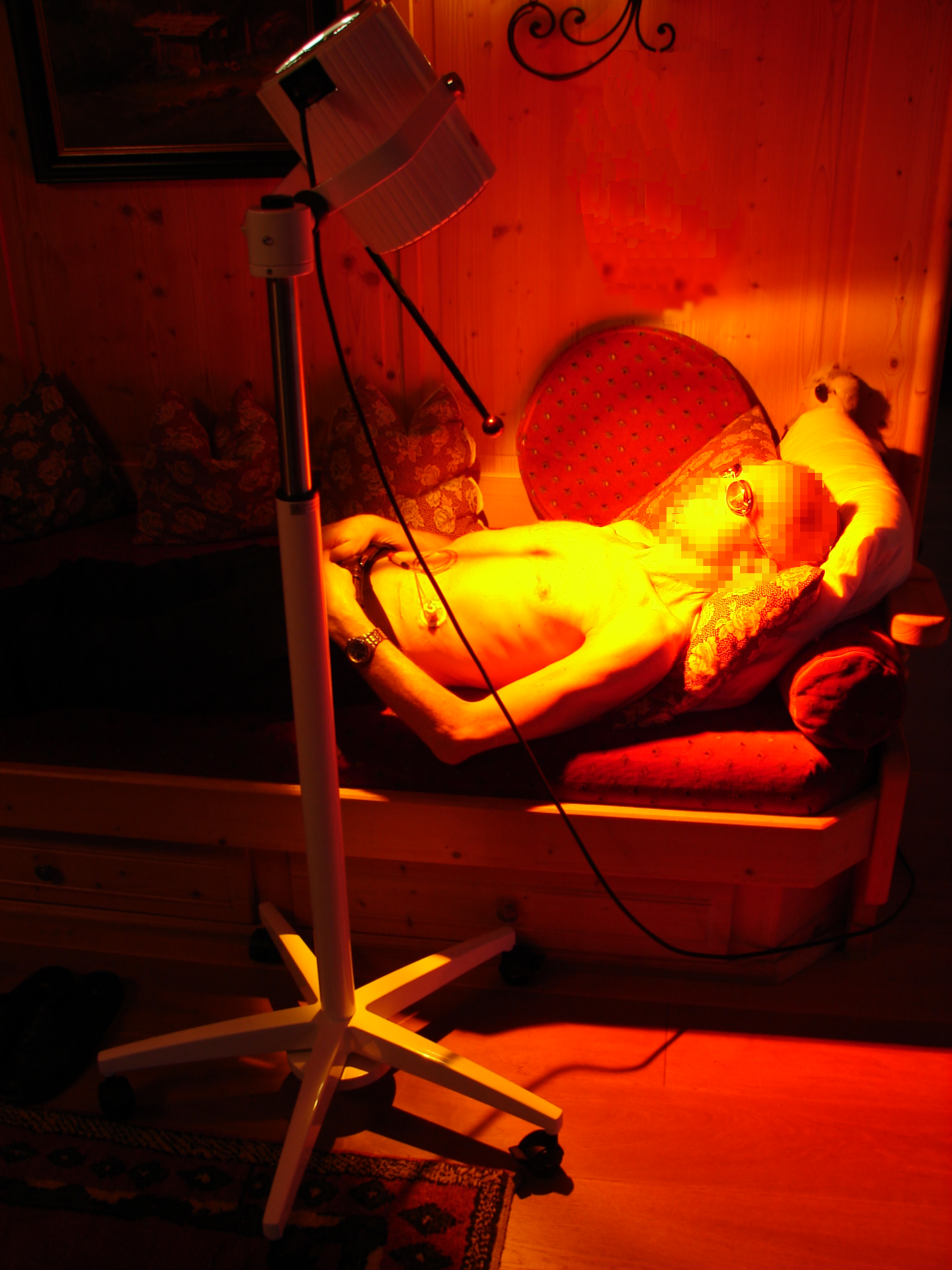
Irradiation with water-filtered infrared-A (wIRA) at home. (A radiator for wIRA emits – besides wIRA – as well visible light.)

At that time, following advices by the author, acetylcysteine (2 times 200 mg per day, given via the PEG tube) and irradiation with water-filtered infrared-A (wIRA) – a special form of heat radiation (Figure 1 (Fig. 1)) [1], [2], [3] – of the ventral part of the neck and the thorax were added to the therapy (manufacturer of the wIRA radiator: Hydrosun, Müllheim, Germany, radiator type Hydrosun 750 FS). Within 1 day with acetylcysteine saliva and mucus became less viscous. Within 2 days with wIRA (one day with 4 to 5 hours with irradiation with wIRA at home; Figure 2 (Fig. 2)), salivation decreased markedly and the patient’s quality of life clearly improved: For the first time the patient slept without interruption and without the need for sleep-inducing medication. After five days with wIRA the patient could eat his first soft dumpling although drinking of fluids was still not possible. After 2½ weeks with wIRA the patient could eat his first minced schnitzel (escalope). The ability to swallow concentrated/incrassated fluids, more in the sense of an eating than a drinking, was regained.

Following the commencement of wIRA (with typically approximately 90–150 minutes irradiation with wIRA per day) the patient had 8 months with good quality of life with only small amounts of liquid saliva and mucus and without the necessity to spit out. During these 8 months the patient was able to sleep during the night. Some weeks after the commencement of wIRA, following consideration by the author, some additional aspects were optimized: As the patient combined oral nutrition intake with PEG tube feeding, a different PEG nutrition was chosen which induces much less viscous saliva. In addition, therapeutic training from a speech therapist having a special education in swallowing disorders was started and performed regularly.

Unfortunately the basic disease progressed – in spite of additional chemotherapy – and the patient died 8 years and 5 months after the operation (8½ months after starting with wIRA).

## Discussion

Water-filtered infrared-A (wIRA) is a special form of heat radiation (in the range 780–1400 nm) with high tissue penetration and a low thermal load to the skin surface (Figure 1 (Fig. 1)) [1], [2], [3], [4], [5]. 

Water-filtered infrared-A is produced by special radiators. The complete broadband radiation of a 3000 Kelvin halogen bulb is passed through a cuvette containing water [2], [6]. The water in the cuvette absorbs or decreases those parts of infrared radiation (most parts of infrared-B and -C and the absorption bands of water within infrared-A), which would otherwise, by reacting with water molecules in the skin, cause an undesired thermal load to the surface of the skin (Figure 1 (Fig. 1)) [1], [4], [7], [8], [9], [10].

The remaining wIRA radiation (in the range 780–1400 nm) has a high penetration capacity in tissue so that in comparison to conventional unfiltered infrared radiation a considerably higher amount of energy can be transferred deeply into the tissue while the thermal load to the skin surface remains low [4], [11], [12]. Thermography shows different skin surface temperature with the same total irradiance: a water-filtered infrared-A radiator causes a lower skin surface temperature than conventional infrared radiators without water-filter [4]. With equal skin surface temperature the total irradiance of infrared-A of a water-filtered infrared-A radiator is nearly 4–9-fold compared to conventional infrared radiators without water-filter [3], [13]. For certain clinically relevant wavelengths, such as 820 nm [14], the irradiance can be even approximately 6–30-fold (see Figure 1 (Fig. 1)) [5], [13], [15], [16]. A typical wIRA radiator emits no ultraviolet radiation (UV) and almost no infrared-B and infrared-C (less than 0.5% compared to 50–80% infrared-B and infrared-C in conventional infrared radiators without water-filter) [4], [5], [11], [12], [13], [15], [16], [17].

wIRA increases tissue temperature (+2.7°C at a tissue depth of 2 cm), tissue oxygen partial pressure (+32% at a tissue depth of 2 cm) and tissue perfusion [3], [18]. 

The 5 main clinical effects of wIRA are: wIRA decreases pain, inflammation and exudation/hypersecretion, and promotes infection defense and regeneration, all in a cross-indication manner [1], [3], [4]. Therefore there is a wide range of clinical indications for wIRA (see e.g. in [2], [19]).

The effects of wIRA are based on both its thermal effects (relying on transfer of heat energy) and thermic effects (temperature-dependent effects, occurring together with temperature changes) as well as on non-thermal and temperature-independent effects like direct effects on cells, cell structures or cell substances [1], [2], [3], [4]. 

Decrease of pain and inflammation and promotion of infection defense and regeneration can be explained both by thermal and non-thermal effects [2], [19]. Concerning decrease of pain by wIRA an increased perfusion allows a better elimination of accumulated metabolites, as pain mediators, lactate, or bacterial toxins, and increases – together with an increased tissue temperature – metabolism (improved metabolisation of accumulated substances and improved regeneration) [18]; non-thermal effects include direct effects on cells and cellular structures and substances and perhaps as well on nociceptors [18]; in addition wIRA relaxes muscles and decreases pain by this as well [19]. Thermal effects include an increased energy production – which is decisive for a variety of processes including regeneration – by higher temperature and higher oxygen partial pressure [1], [2]. 

A decrease of exudation/hypersecretion by wIRA is observed e.g. as well in wounds [3], [5] and can be explained by non-thermal effects [2], [19].

Concerning non-thermal effects the energy-rich wavelengths near to visible light – approximately 780–1000 nm (800–900 nm [20], [21], [22], 800 nm [23], 820 nm [14], 830 nm [24]) – seem to be the clinically most important part of wIRA [2], [5]. 

Non-thermal effects include an influence on the cytochrome C oxidase in the mitochondria: cytochrome C oxidase is known as universal photo acceptor for radiation of approximately 600–1000 nm with absorption maxima at 620, 680, 760 and 825 nm [25], [26], [27]. By absorption of radiation the cytochrome C oxidase can induce signalling cascades and therefore has regulatory function far beyond energy production, described in detail in [2], based on [14], [25], [26], [27].

In the patient case mentioned above the decrease of hypersecretion (hypersalivation) and the overcoming of a swallowing disorder (interpreted for most of the time span more as a problem of choking in the sense of a functional dysphagia rather than as a swallowing disorder from a structural stenosis; swallowing as a complex nerve function), possibly interpretable as regeneration of nerve function, were the two most important underlying effects of wIRA concerning improving his quality of life. 

The patient clearly benefited from some general features of wIRA, as mentioned e.g. in [1], [2], [3], [5]: All irradiations of the patient with wIRA were done at home and were contact-free without the use of expendable materials and were felt to be pleasant. A moderate irradiance was always used by choosing enough distance between radiator and uncovered skin (see Figure 2 (Fig. 2)), approximately two times the length of the distance rod (distance rod = minimum irradiation distance). After receiving instructions in proper and safe use of wIRA the patient could easily apply wIRA at home by himself. This allowed long daily irradiation times and use of wIRA even at weekends and avoided the necessity of visiting a physician or a physiotherapist with a wIRA radiator for each treatment, thereby saving both time and money.

Even bronchial hypersecretion can be markedly decreased by thoracic irradiation with wIRA. Pulseoximetrically measured haemoglobin oxygen saturation, in another patient, increased reproducibly within approximately 20–30 minutes without taking other actions (especially without tracheal suction) from 90 to 95% [2].

As wIRA can improve the regeneration of nerve function, wIRA can be used to treat polyneuropathia (polyneuropathia with unknown reason or polyneuropathia induced e.g. by chemotherapy) and to decrease paresthesia markedly [2], [19].

It should be emphasized that in this case report wIRA was used only symptomatically and not as part of a causal therapy, although wIRA can be combined successfully in oncology with radiation therapy or with chemotherapy [28], [29], [30], [31], [32].

The case description is in accordance with the assessment of a palliative care ward of another university hospital that water-filtered infrared-A (wIRA) can cause impressive positive effects especially in patients with irradiated head and neck tumors.

## Conclusion

Besides in a variety of other indications for wIRA, in cases of swallowing disorders (functional dysphagia) and hypersalivation or hypersecretion of mucus the use of wIRA should be considered as part of the treatment regime for improving a patient’s quality of life.

## Notes

### Competing interests

The author is working for the Dr. med. h.c. Erwin Braun Foundation, Basel, a charitable, non-profit Swiss scientific foundation approved by the Swiss Federal Administration. The foundation supports clinical investigation of water-filtered infrared-A. The foundation was not involved in any content- or decision-related aspect of the case report. The author is not and was not employed by a commercial company or received fees or grants by a commercial company in the field of water-filtered infrared-A. Therefore, the author declares that no conflicts of interest exist according to the guidelines of the International Committee of Medical Journal Editors.

### Ethical statement

Both the patient and his wife gave informed consent for the publication of a case report including the picture of the irradiation at home.
